# Supplementary material for: MetaRibo-Seq measures translation in microbiomes
Source: Nat Commun. 2020 Jun 29;11:3268. doi: 10.1038/s41467-020-17081-z (PMC7324362; doi:10.1038/s41467-020-17081-z)
Supplement: Supplementary file 10 — Supplementary Data 7 [file 41467_2020_17081_MOESM10_ESM.zip › File2/Confidence_VeryHigh_Taxonomy/2053_out.krona.html]

Javascript must be enabled to view this page.

members
magnitude
magnitudeUnassigned
count
unassigned
taxon
rank

2053\_out

35

35
superkingdom
2

35
1239
phylum

35
class
186801

35
order
186802

35
family
186803

841
genus
35

species
1897015

SRS016095\_contig\_number\_27923
1

species
2292367
1

SRS013951\_contig\_number\_contig-100\_285.214110

3

SRS050998\_contig\_number\_15232SRS064645\_contig\_number\_contig-100\_398.398SRS144362\_contig\_number\_44406
species
360807

166486
species
30

SRS013158\_contig\_number\_4192SRS014613\_contig\_number\_22094SRS015065\_contig\_number\_contig-100\_273.218031SRS015578\_contig\_number\_29662SRS015579\_contig\_number\_25431SRS015960\_contig\_number\_19149SRS016335\_contig\_number\_3604SRS018817\_contig\_number\_contig-100\_2717.116868SRS019068\_contig\_number\_113247SRS024549\_contig\_number\_5253SRS052027\_contig\_number\_12146SRS054956\_contig\_number\_10781SRS056519\_contig\_number\_16320SRS058070\_contig\_number\_contig-100\_897.185582SRS075341\_contig\_number\_16146SRS075716\_contig\_number\_contig-100\_295.43768SRS076929\_contig\_number\_contig-100\_2323.137589SRS098061\_contig\_number\_3469SRS098827\_contig\_number\_13093SRS098881\_contig\_number\_27786SRS1041130\_contig\_number\_4322SRS104485\_contig\_number\_4288SRS104975\_contig\_number\_2477SRS105153\_contig\_number\_contig-100\_672.230067SRS143417\_contig\_number\_19197SRS143780\_contig\_number\_32940SRS148091\_contig\_number\_10881SRS148159\_contig\_number\_41384SRS149879\_contig\_number\_26401SRS893378\_contig\_number\_contig-100\_107.143125
